# Fluorine ion induced phase evolution of tin-based perovskite thin films: structure and properties†

**DOI:** 10.1039/c9ra07415e

**Published:** 2019-11-13

**Authors:** Junsheng Wu, Fang Fang, Zhuo Zhao, Tong Li, Rizwan Ullah, Zhe Lv, Yanwen Zhou, David Sawtell

**Affiliations:** School of Chemical Engineering, University of Science and Technology Liaoning 114051 Liaoning Anshan China; Institute of Surface Engineering, University of Science and Technology Liaoning Anshan 114051 Liaoning China zhouyanwen@ustl.edu.cn; Department of Physics, Beijing Normal University 100875 Beijing China; Surface Engineering Group, Manchester Metropolitan University Manchester M1 5GD England UK d.sawtell@mmu.ac.uk

## Abstract

To study the effect of fluorine ions on the phase transformation of a tin-based perovskite, CsSnI_3−*x*_(F)_*x*_ films were deposited by using thermal vacuum evaporation from a mixed powder of SnI_2_, SnF_2_ and CsI, followed by rapid vacuum annealing. The color evolution, structure, and properties of CsSnI_3−*x*_F_*x*_ films aged in air were observed and analyzed. The results showed that the colors of the films changed from black to yellow, and finally presented as black again over time; the unstable B-γ-CsSnI_3−*x*_F_*x*_ phase transformed into the Y-CsSnI_3−*x*_F_*x*_ phase, which is then recombined into the Cs_2_SnI_6−*x*_F_*x*_ phase with the generation of SnO_2_ in air. Fluorine dopant inhibited the oxidation process. The postponement of the phase transformation is due to the stronger bonds between F and Sn than that between I and Sn. The color changing process of the CsSnI_3−*x*_F_*x*_ films slowed that the hole concentrations increased and the resistivities decreased with the increase of the F dopant ratio. With the addition of SnF_2_, light harvesting within the visible light region was significantly enhanced. Comparison of the optical and electrical properties of the fresh annealed CsSnI_3−*x*_F_*x*_ films showed that the band gaps of the aged films widened, the hole concentrations kept the same order, the hole mobilities reduced and therefore, the resistivities increased. The double layer Cs_2_SnI_6−*x*_F_*x*_ phase also showed ‘p’ type semi-conductor properties, which might be due to the incomplete transition of Sn^2+^ to Sn^4+^, *i.e.* Sn^2+^ provides holes as the acceptor.

## Abstract 2

Fluorine dopant inhibited the oxidation process in CsSnI_3−*x*_F_*x*_ films with high carrier concentration, low resistivity and wide light absorption.

## Graphical abstract

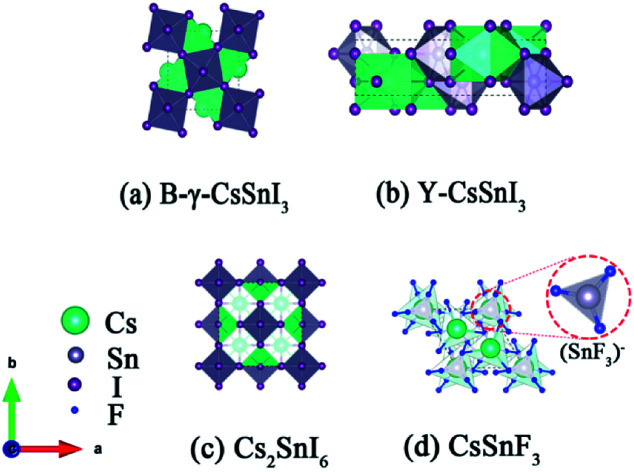

## 1. Introduction

Within the past decade, inorganic–organic hybrid perovskite solar cells have drawn researchers' attention for their enhanced efficiencies due to their superior long diffusion length, with a conversion efficiency of approximately 3.8% in 2009 ^1^ to greater than 23.32% today.^2^ Since the early reports of lead (Pb)^3^ and tin (Sn)^4^ based perovskite solar cells, the efficiencies of Pb based perovskite solar cells have increased to nearly 22%, which exceeds those of poly-silicon solar cells.^5^ Although most studies are focused on MAPbX_3_ perovskite materials, tin-based perovskite has attracted more and more attention due to the low toxicity of tin.^6^ So far, the literature^7,8^ has reported that both Sn doped and the entirely Sn based perovskite solar cells (ASnX_3_) are not as efficient as Pb-based perovskite solar cells due to two primary reasons: (1) tin is easily oxidized from Sn^2+^ to Sn^4+^ oxidation states whilst exposed to air.^9,10^ The diffusion length of the photogenic carriers is limited because of too many p-type carriers produced by the self-doping effect within the tin-based perovskite materials; (2) SnI_2_ and MAI (CH_3_NH_3_I^−^) reacts quickly and rapidly crystallizes during spin-coating process^9,11^ and therefore, it is difficult to control the crystallinity of perovskite, lead to poor coverage and uniformity of the film. To combat this stable and defect free ASnX_3_films are required.

**Fig. 1:**
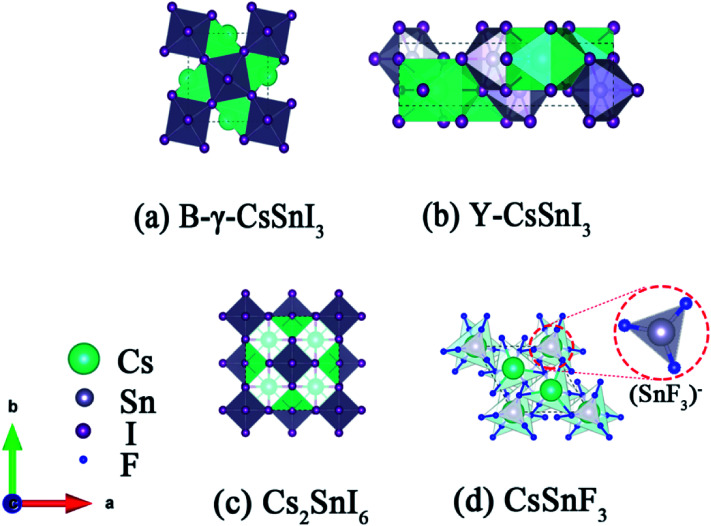
Polyhedral model of (a) B-γ-CsSnI_3_. (b) Y-CsSnI_3_. (c) Cs_2_SnI_6_ and (d) CsSnF_3_. SnI_6_ octahedra are in gray, Cs is in green, Sn is in gray, the I anions are purple, and F is blue.

Inorganic materials generally have higher stability than organic materials. For this reason, the studies on inorganic perovskite materials have been proposed. As the standard ASnX_3_ perovskite, the schematic crystal structure of B-γ-CsSnI_3_, Y-CsSnI_3_ and Cs_2_SnI_6_ are shown in Fig. 1, in which the B-γ-CsSnI_3_ phase is black with a three dimensional perovskite structure,^12^ the Y-CsSnI_3_ phase is yellow with a one-dimensional double-chain structure,^13^ and Cs_2_SnI_6_ is black with double layer perovskite structure. The unstable B-γ-CsSnI_3_ phase promptly transforms to the Y-CsSnI_3_ phase, then forms into the Cs_2_SnI_6_ phase in air, accompanied with the formation of SnO_2_. The compound of Cs_2_SnI_6_ exhibits its stability in damp air due to the stable Sn^4+^ state. The ‘vacancy ordered’ double layer perovskite structure is formed by the missing half of the Sn atoms located in the center of octahedral, and then reconstitutes to the discontinuous regular octahedral structure (SnI_6_).^2–14^

ASnX_3_ perovskite structure (A = metal or NH_4_ and X = halogen family) generally consists of (SnX_3_)^−^ and M^+^, in which Sn^2+^ is unstable. Vilminot originally discussed evolution of the ionic-conductivity of the MSnF_3_ phases (M = Na, K, Rb, Cs, NH_4_, Tl) in 1985.^15^ For example, CsSnF_3_ exhibits a crystal structure consisting of isolated (SnF_3_)^−^ anionic polyhedral,^16^ shown in Fig. 1d. Each Sn^2+^ cation in CsSnF_3_ is bonded to three fluorine atoms in a distorted triangular pyramidal coordination environment, *i.e.* the lone pair of 5s electrons of tin(ii) and three pairs of electrons, shared unequally with the fluorine ions, occupied on the most stable configuration sp^3^ hybridization orbits.^17^

Researchers and experiments have shown that the tolerance factors of the stable perovskite materials should be between 0.78–1.05,^18^ in which the tolerance factor, *t* = (*R*_A_ + *R*_X_)/√2(*R*_B_ + *R*_X_). In the formula, *R*_A_ is the radius of monovalent cation, *R*_B_, the radius of divalent metal cations and *R*_X_, the radius of the halide ion. If there are multiple ions in the A and/or B positions, the average radius should be taken. The perovskite structure tends to be stable when *t* is close to 1. By calculation, the ‘*t*’ of CsSnI_3−*x*_F_*x*_ is within 0.8734–0.9587, and *t* gets larger as the F ratio increases. That means that the perovskite structure of CsSnF_3_ is more stable than that of CsSnI_3_, and F doped in CsSnI_3_ may delay the phase transformation of CsSnI_3_.

The crystal parameters of the CsSnI(F)_3_ phases are shown in Table 1. The Sn–I bond length is stretched whilst the B-γ-CsSnI_3_ phase transformed into Y-CsSnI_3_, and then shortened along with the initial formation of the stable Cs_2_SnI_6_ during the process of tin oxidization. There are many factors affecting bond length and bond energy, such as atomic radius, distance between nuclei, repulsive force between lone pair electrons, feedback bond and so on. In ionic compound, ion radius is the main contributor to bond length. Therefore, the Sn–F bond length in CsSnF_3_ is less than the bond length of Sn–I in CsSnI_3_, the difference between them is 1.1321 Å due to the much smaller ionic radii of F (1.33 Å) compared to I (2.20 Å). Generally, the order of stability of the halide complexes of tin(ii) is F > Cl > Br > I,^17^ tin(ii) preferentially bonds with fluoride ion. Hence, SnF_2_ was introduced as the doping source to improve the stability of tin-based perovskite materials. Meanwhile, as an antioxidant, fluoride ion inhibits the oxidation process of Sn^2+^,^10,19^ the fluorine also reduces the densities of the materials, which improves their performance when used as a photovoltaic material.^20^

**Table 1:** Crystallographic refinement details for the tin based perovskite polymorphs

Sample	Type	Bond length (Å)	Lattice constant (Å per °)				Ref.
B-γ-CsSnI_3_	Cs–I	4.09495	*a*	8.6885	*α* = *β* = *γ*	90	38
			*b*	12.3775			
	Sn–I	3.1685	*c*	8.6384			
Y-CsSnI_3_	Cs–I	4.0680	*a*	10.350	*α* = *β* = *γ*	90	38
	Sn–I	3.2475	*b*	4.7632			
			*c*	17.684			
Cs_2_SnI_6_	Cs–I	4.2671	*a* = *b* = *c*	11.6276	*α* = *β* = *γ*	90	6
	Sn–I	2.9107					
CsSnF_3_	Cs–F	3.06629	*a* = *b*	7.18763	*α* = *β*	90	43
	Sn–F	2.11543	*c*	16.08594	*γ*	120	
CsSnI_3−*x*_F_*x*_ (non-optimized)	Cs–I	4.0215	*a*	8.688	*α* = *β* = *γ*	90	
	Sn–I	3.1143	*b*	12.378			
	Cs–F	3.3794	*c*	8.6430			
	Sn–F	3.2934					

Even though the main method to prepare CsSnI_3_ films is still the one-step solution based process, it is difficult to produce dense, pinhole free film due to the rapid crystallization of tin-based perovskite.^9,20–22^ The films prepared by this method are very sensitive to film formation conditions, such as annealing temperature,^23,24^ solution concentration,^25,26^ precursor solution composition^27,28^ and solvent selection.^29–31^ Due to the evaporation of the solvent and the volatilization of the materials during the process of annealing, the crystal tends to easily aggregate and shrink, and the morphology of the films are mainly cluster-like and needle-like, so it is easy to cause the devices' efficiencies to be uneven. The thermal vacuum evaporation technique is an effective approach to prepare high coverage homogeneous thin films and has been widely used in lead-based perovskites.^32,33^ Therefore, tin-based perovskite films should be possible to be prepared by this technique.^10,34^ Here, the mixture powder of SnI_2_ and CsI were evaporated onto the glass slide substrates by thermal vacuum evaporation method to form fully covered, dense, pinhole free CsSnI_3_ perovskite film. Also, by adding SnF_2_ powder into the mixture, the CsSnI_3−*x*_F_*x*_ films were prepared as well. The evolution of the color, structure and properties of the annealed freshly and annealed aged CsSnI_3−*x*_F_*x*_ films over time was observed, measured and analyzed to explore the effect of F doping.

## 2. Experimental details

### 2.1. Sample preparation

Cesium iodide (99.9% CsI), tin(ii) fluoride (99.99% SnF_2_) and tin iodide (99.9% SnI_2_) were produced by Ying Kou You Xuan Trade Co., Ltd. China. Acetone (99.7%) and ethanol (99.7%) were purchased from Sinopharm Chemical Reagent Co., Ltd. China. Deionized water was filtered in the laboratory. All the reagents were of analytical grade and used as received.

The process of sample preparation was shown in Fig. S1.† Weighted SnI_2_, SnF_2_ and CsI powder was mixed in a mortar and placed in a tungsten boat of size 50 × 15 × 2 mm, refer to Table 2. The AC power (50 Hz frequency) was applied to the tungsten boat through two connected electrodes, the glass slides, CAT. no. 7101 with a size of 25.4 × 76.2 mm, were ultrasonically cleaned in acetone for 900 s, diluted by deionized water and ethanol before being loaded into the DM 450C vacuum chamber. The glass slide was held above the tungsten boat at a separation of 150 mm. The chamber was pumped down to 2 × 10^−3^ Pa and the SnI_2_, SnF_2_ and CsI was evaporated at a voltage of 70 V and current of 140 A. The evaporation process referred to Tong.^35^ The films were finally annealed at 210 °C for 4 min in argon gas by the Rapid Thermal Processor of RTP-500V.

**Table 2:** The chemical composition of CsSnI_3−*x*_F_*x*_ thin films

Sample	CsI (mol)	SnI_2_ (mol)	SnF_2_ (mol)	Proportion
CsSnI_3_	0.0025	0.0025	0	100 : 100 : 0
CsSnI_2.88_F_0.11_	0.0025	0.0024	0.000094	100 : 96.25 : 3.75
CsSnI_2.78_F_0.22_	0.0025	0.0023	0.000190	100 : 92.5 : 7.5
CsSnI_2.67_F_0.33_	0.0025	0.0022	0.000280	100 : 88.75 : 11.25

### 2.2. Measurement techniques

The thicknesses of the films were measured by using a KLA-Tencor Alpha-step D-100 type profilometer on a step created on the films by masking the glass substrates. The phases of the CsSnI_3−*x*_F_*x*_ films were measured by X'Pert powder X-ray diffractometer (XRD) in glancing angle scanning mode at 0.5° incident angle with Cu Kα X-ray from 10° to 80°, and analyzed by High-Score software.^36^ The electrical and optical properties of the CsSnI_3−*x*_F_*x*_ films were measured by a HALL 8800 Hall Effect Measurement device and a CARY 5000 UV-Vis-NIR spectrometer over the wavelength range of 300 to 1000 nm, respectively. AFM tests were performed in ambient conditions at room temperature with scanning probe microscopy (Bruker, Multimode 8 with controller V). A Pt/Ir-coated tip on a Si cantilever (tip radius of 20 nm, force constant of 2.8 Nm^−1^ and a resonant frequency of 75 kHz) was used to characterize the topography of the films. The typical tip-scanning velocity was 2 μm s^−1^.

## 3. Results & discussion

### 3.1. Morphological structure

**Fig. 2:**
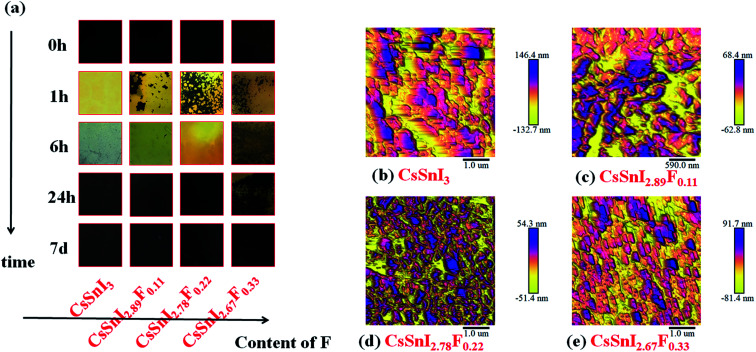
(a) Macroscopic images of the evolution in the cesium tin iodine doped fluorine (CsSnI_3−*x*_F_*x*_) films. (b–e) AFM images of the cesium tin iodine doped fluorine (CsSnI_3−*x*_F_*x*_) with different fluorine content, (b–e) correspond to CsSnI_3_, CsSnI_2.89_F_0.11_, CsSnI_2.78_F_0.22_, CsSnI_2.67_F_0.33_, respectively.

To demonstrate the macro-evolution process of the fluorine doped cesium tin iodine (CsSnI_3−*x*_F_*x*_) films, photographs of the colors of the freshly annealed and aged CsSnI_3−*x*_F_*x*_ films exposed to ambient air were shown in Fig. 2a. With increasing time, the colors of the films changed from black to yellow, and finally presented as black again. These colors served as a good indication of the oxidation progress of B-γ/Y-CsSnI_3_ to Cs_2_SnI_6_ in air.^37^ It is evident from a comparison of the photographs in Fig. 2a that doping F slowed down the transformation process of the perovskite phases, since the color of F doped films were predominantly yellow after six hours exposure in air, whereas that of the CsSnI_3_ film without fluorine dopant had almost completely blackened. The morphologies of the corresponding CsSnI_3−*x*_F_*x*_ films were examined by AFM, respectively (see Fig. 2b–e). Strikingly, no pinhole appeared in entire scope in the CsSnI_3−*x*_F_*x*_ films. Furthermore, the grain sizes became finer as the amount of doped fluorine increased.

### 3.2. Phase structure

**Fig. 3:**
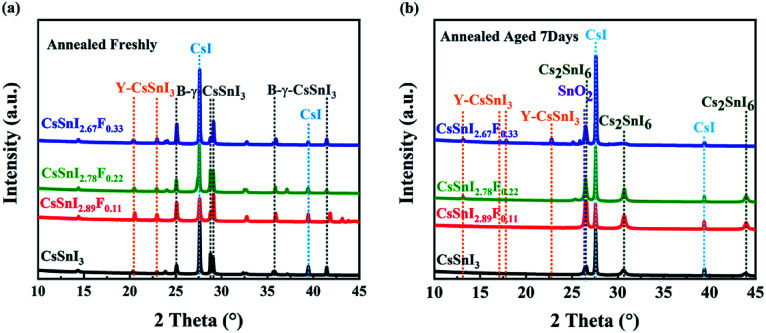
Evolution of XRD patterns of CsSnI_3−*x*_F_*x*_ films with time. (a) XRD patterns of the CsSnI_3−*x*_F_*x*_ films annealed freshly, (b) XRD patterns of the annealed CsSnI_3−*x*_F_*x*_ films exposed in air for 7 days.

Corroborating evidence for the process of phase transition in CsSnI_3−*x*_F_*x*_ films \exposed in air was provided by X-ray diffraction (XRD), shown in Fig. 3a and b, which were consistent with the variations in morphology. Firstly, for the annealed CsSnI_3−*x*_F_*x*_ films (see Fig. 3a), the peaks at 25.11°, 29.08° and 41.45° were attributed to B-γ-CsSnI_3_ phase (022), (220) and (224) planes (*Ref.Code*: 01-043-1162), respectively. Meanwhile, the well-matched peaks of 27.61° and 39.44°, assigned to CsI phase (110) and (200) planes (*Ref.Code*: 01-077-2185), were observed. However, the annealed XRD patterns also showed Y-CsSnI_3_ phase (111) and (121) planes (*Ref.Code*: 01-071-1898). The B-γ-CsSnI_3_ peaks of the annealed CsSnI_3−*x*_F_*x*_ films became more distinct with the increase of F dopant ratios whilst the Y-CsSnI_3_ peaks became weaker. Although stoichiometric ratios were strictly controlled, the sharp and high intensity peaks of CsI also appeared in the XRD patterns, this can be attributed to incomplete reaction with SnI_2_, because SnI_2_ (vaporization point 993.15 K in ambient air) is easier to evaporate and decompose (unstable Sn^2+^) than CsI (vaporization point 1553 K in ambient air) in the process of thermal evaporation. The variations of the XRD patterns show that fluorine doping obstructed the transformation from B-γ-CsSnI_3_ to Y-CsSnI_3_ perovskite phases.

After aging seven days in air, the films returned to a light black color. This phenomenon is consistent with the trend of Qiu's experiment.^37^ The new diffraction peaks (see Fig. 3b) at 26.44°, 30.61°, and 43.91° were attributed to the (222), (004) and (044) orientations of Cs_2_SnI_6_ phase (*Ref.Code*: 00-051-0466). However, the aged XRD patterns showed (120), (121), (130) and (210) planes of the Y-CsSnI_3_ phase. Furthermore, several diffraction peaks at 27.57°, 39.41°, and 26.58° assigned to CsI and SnO_2_ (*Ref.Code*: 00-077-0448) were observed as well. Again, the Y-CsSnI_3_ phase was much obvious with the increase of F dopant ratios, which meant F doping delayed the phase transformation, and Y phase existed in the highly F doped films even after aging for seven days.

Based on these findings *via* the above XRD data, partial CsI, SnI_2_, and SnO_2_ peaks were observed during the evolution process of the CsSnI_3−*x*_F_*x*_ films, the chemical reactions involved may be as follows:
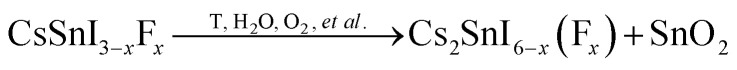


Therefore, it is clear that this process was due to oxidation of Sn in the compounds.^6^ Sn^2+^ ions are quite sensitive to external oxygen, especially in humid environments, which can be oxidized to more stable Sn^4+^ analogues. This oxidation process may fundamentally destroy the charge neutrality of the CsSnI_3_ perovskite structure and lead to phase transition. The Sn–F bonds form when fluorine replaces iodide in the lattice. The Sn–F bond length is shorter than that of Sn–I, resulting in a more stable crystal structure and weakening the role of oxygen, refer to Fig. S2.† In short, doping with fluorine slowed down the oxidation progress of the Sn^2+^ in CsSnI_3_ into Sn^4+^. The B-γ-CsSnI_3−*x*_(F)_*x*_ phase eventually transferred into Cs_2_SnI_6−*x*_(F)_*x*_ and was accompanied by the formation of SnO_2_ over time, but the formation processes are delayed by the additional F dopant. The remnant Y-CsSnI_3_ phase proved not only to delay the phase transition, but also the existence of Sn^2+^. The hybrid of Sn^4+^with Sn^2+^ resulted in the Cs_2_SnI_6−*x*_(F)_*x*_ films showing their p type property.

### 3.3. Electrical properties of CsSnI_3−*x*_F_*x*_ films

The electrical properties and thickness of the CsSnI_3−*x*_F_*x*_ films at room temperature were measured by a HALL 8800 Hall Effect Measurement device and KLA-Tencor Alpha-step D-100 type profilometer respectively. The results were shown in Table 3 for the freshly annealed and Table 4 for those aged seven days after annealing. In this case, the thicknesses of the films were used to calculate the resistivity of the films. Both the annealed and aged CsSnI_3−*x*_F_*x*_ films were p type semiconductors with the holes provided by the Sn vacancies. Intrinsic defects such as Sn vacancies in the ternary Cs–Sn–I system gave rise to p-type conductivity^20^ and DFT calculations have shown that the formation energy of V_Sn_ defects was the lowest among all defects.^38^ In Table 3, the freshly annealed CsSnI_3_ film exhibited the carrier densities of ∼10^14^ cm^−3^, and the carrier density of CsSnI_3−*x*_F_*x*_ films increased to ∼10^16^ cm^−3^ with the increase of SnF_2_ content. It's also worth noting that the resistivities of CsSnI_3−*x*_F_*x*_ films were 1–2 orders of magnitude smaller than that of undoped film. The reduction of the Sn vacancy concentrations can be attributed to the strong bonding energy between F and Sn.

**Table 3:** The electrical properties and thickness of CsSnI_3−*x*_F_*x*_ thin films annealed freshly

Sample	*R*_s_ (Ω □^−1^)	*ρ* (Ω cm)	N/P (cm^−3^)	*μ* (cm^2^ V^−1^ s^−1^)	Thickness (nm)	Type
CsSnI_3_	2.14 × 10^5^	22.71	6.12 × 10^14^	449.31	1060	P
CsSnI_2.88_F_0.11_	1.93 × 10^4^	4.65	5.84 × 10^15^	229.55	2410	P
CsSnI_2.78_F_0.22_	4.99 × 10^3^	1.44	6.01 × 10^16^	226.67	2880	P
CsSnI_2.67_F_0.33_	1.49 × 10^3^	0.394	7.29 × 10^16^	217.34	2650	P

**Table 4:** The electrical properties and thickness of CsSnI_3−*x*_F_*x*_ thin films annealed aged 7 days in air

Sample	*R*_s_ (Ω □^−1^)	*ρ* (Ω cm)	N/P (cm^−3^)	*μ* (cm^2^ V^−1^ s^−1^)	Thickness (nm)	Type
CsSnI_3_	3.62 × 10^5^	38.75	1.42 × 10^15^	114.96	1060	P
CsSnI_2.88_F_0.11_	4.07 × 10^4^	9.79	8.55 × 10^15^	74.55	2410	P
CsSnI_2.78_F_0.22_	1.44 × 10^4^	4.16	6.21 × 10^15^	241.46	2880	P
CsSnI_2.67_F_0.33_	3.54 × 10^5^	93.75	1.45 × 10^16^	4.59	2650	P

After aging in air for seven days, the electrical properties of the CsSnI_3−*x*_F_*x*_ films kept the same order. As described in Section 3.2 Phase structure, the main composition of the CsSnI_3−*x*_F_*x*_ films was Cs_2_SnI_6_. This is accompanied by the generation of a mass of Sn vacancies. The carrier densities of the aged CsSnI_3−*x*_F_*x*_ films were almost at the same level in comparison to those of the annealed CsSnI_3−*x*_F_*x*_ films. The resistivities of CsSnI_3−*x*_F_*x*_ films were still relatively small. After aging for seven days, the phase transformation occurred, the oxidation of tin was completed, and the double layer ‘vacancy ordered’ phase Cs_2_SnI_6−*x*_F_*x*_ formed. As the results, the Sn vacancies of the aged films were high, and their carrier density increased sharply. However, the rate of increase of the charge carriers of the aged films decreased with the increase of SnF_2_ content. The phases were stable due to F doping, the creation processes of the Sn vacancies were delayed and therefore, the changes of the electrical properties were slower. This fully illustrates SnF_2_ hindering the oxidation process of Sn^2+^, which blocked the formation of Cs_2_SnI_6_.

Since the carrier (hole or electron) concentration of semiconductor depends on the inherent defect concentration, the control of carrier concentration is a necessary condition to optimize the performance of solar cells. It works better as the hole-transport material (HTM) when hole concentration and conductivity are high, but it may work better as the light absorber material (LAM) when hole concentration and conductivity are moderate.^39^ From the data of electrical properties, by tuning the F doping amount, hole concentration and conductivity can be controlled, so that the film can be selectively arranged as HTM and LAM.

### 3.4. Optical properties

**Fig. 4:**
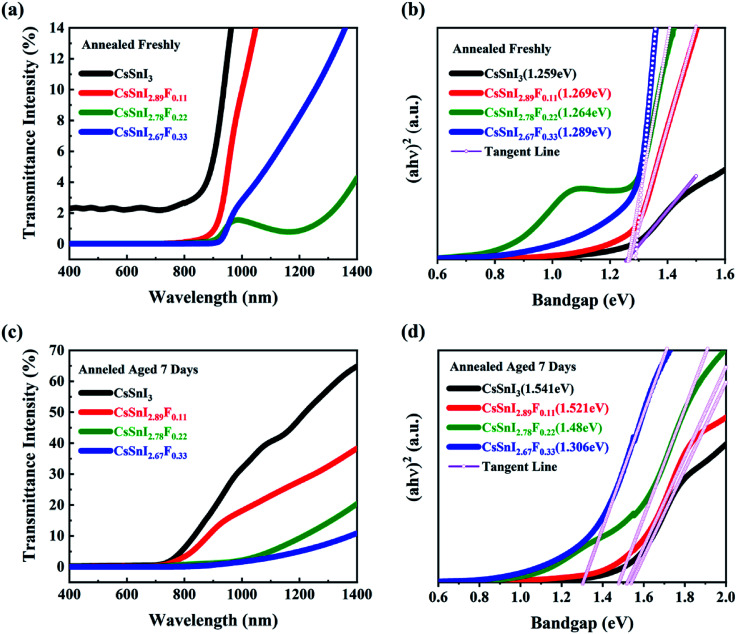
Optical spectra of CsSnI_3−*x*_F_*x*_ films:(a and b) annealed freshly; (c and d) under exposure to ambient air for 7 days.

The absorptive spectra of the annealed and aged CsSnI_3−*x*_F_*x*_ films within the range of 400–1400 nm were presented in Fig. 4a and c, respectively. Within the UV range, the absorptivity of the films was almost the same. The absorption edges of the annealed and aged CsSnI_3−*x*_F_*x*_ films exhibited obvious differences. With the addition of SnF_2_, the light harvesting in the visible light region of the solar spectrum of the CsSnI_3−*x*_F_*x*_ films was significantly enhanced. These differences indicate the difference of the band gap between samples. The spectra of the absorption *via* optical band gaps of the annealed and aged films were shown in Fig. 4b and d, which were calculated by the formula of *αhθ* = *A*(*hθ* − *E*_g_)^1/2^. The Tauc plot^40^ is used to evaluate the optical energy gap. The bandgaps of all the CsSnI_3−*x*_F_*x*_ films are between 1.25 eV and 1.3 eV as shown in Fig. 4b which was consistent with those previously reported.^41^

The optical spectra of CsSnI_3−*x*_F_*x*_ films under exposure to ambient air for seven days are shown in Fig. 4c and d. The absorption edges of all the CsSnI_3−*x*_F_*x*_ films exhibited blue shift. All the bandgaps for the F doped films got smaller, and was close to 1.48 eV derived in Qiu's report.^37^ This is a result of SnF_2_ eliminating the formation of weak unidentified reflections due to the Y-CsSnI_3_ phase.^42^ After the CsSnI_3−*x*_F_*x*_ films under exposure to ambient air for seven days, the Y-CsSnI_3_ phase only was shown in the highly F doped films, which the fully illustrates the role of SnF_2_. The transmittance of the Cs_2_SnI(F)_6_ films within visible wavelength were strongly affected by doping F ions due to the decomposition of CsSnI_3−*x*_F_*x*_. It was further shown that SnF_2_ blocked the formation of Cs_2_SnI_6_ by the changes of bandgap. Note that the sunlight absorption in the red and near-infrared regions has been a challenge for solar cells, and the excellent optical properties of CsSnI_3−*x*_F_*x*_ films made up for this defect.

## 4. Conclusion

CsSnI_3_ transformed into stable double layer perovskite Cs_2_SnI_6_ phase in air was investigated for potential solar cell applications. By conducting a series of experiments, it was determined that the phase transformation in air can be slowed down by doping F ions into CsSnI_3_. The mechanism of phase transition delay is that Sn^2+^ preferentially bonds with F^−^, and thus the process of Sn^2+^ in CsSnI_3_ to be oxidized into Sn^4+^ was slowed down. The B-γ-CsSnI(F)_3_ phase eventually formed Cs_2_SnI(F)_6_ accompanied by the formation of the SnO_2_ phase over time, but the transition processes are delayed by the additional F dopant.

Furthermore, SnF_2_ improved the carrier concentration and conductivity of the film, in which the resistivities of CsSnI_3−*x*_F_*x*_ films were less than 10 Ω cm except CsSnI_2.67_F_0.33_. The CsSnI_3−*x*_F_*x*_ films also absorbed more visible and infrared light with increased F doping than the pure CsSnI_3_ film. It was further shown that SnF_2_ blocked the formation of Cs_2_SnI_6_ by comparing the changes of bandgaps. The usage of CsSnI_3−*x*_F_*x*_ films as HTM or LAM in solar cells is envisioned to be a promising method of improving efficiency.

## Conflicts of interest

There are no conflicts to declare.

## Supplementary Material

RA-009-C9RA07415E-s001
