# Supplementary material for: Fluorine ion induced phase evolution of tin-based perovskite thin films: structure and properties
Source: RSC Adv. 2019 Nov 13;9(63):37119–26. doi: 10.1039/c9ra07415e (PMC9075532; doi:10.1039/c9ra07415e)
Supplement: RA-009-C9RA07415E-s001 [file RA-009-C9RA07415E-s001.pdf]

Electronic Supplementary Material (ESI) for RSC Advances.
This journal is © The Royal Society of Chemistry 2019

Fluorine Ion Induced Phase Evolution of Tin-based Perovskite Thin Films: Structure and Properties

Junsheng Wu[a], Fang Fang[a], Zhuo Zhao[a], Tong Li[a], Rizwan Ullah[b], Zhe Lv[a],

Yanwen Zhou[a], David Sawtell[c]**

[a] *Institute of Surface Engineering, University of Science and Technology Liaoning, 114051 Liaoning Anshan, China*

[b] *Department of Physics, Beijing Normal University, 100875 Beijing, China*

[c] *Surface Engineering Group, Manchester Metropolitan University, Manchester M1 5GD, England*

***Corresponding author, Email: zhouyanwen@ustl.edu.cn, d.sawtell@mmu.ac.uk.**

Supporting information available:

1. Schematic illustration of the evaporation method

2. Supercells and F doping models of B-γ-$CsSnI_3$

3. Density of Localized Trapped Charge

Schematic illustration of the evaporation method

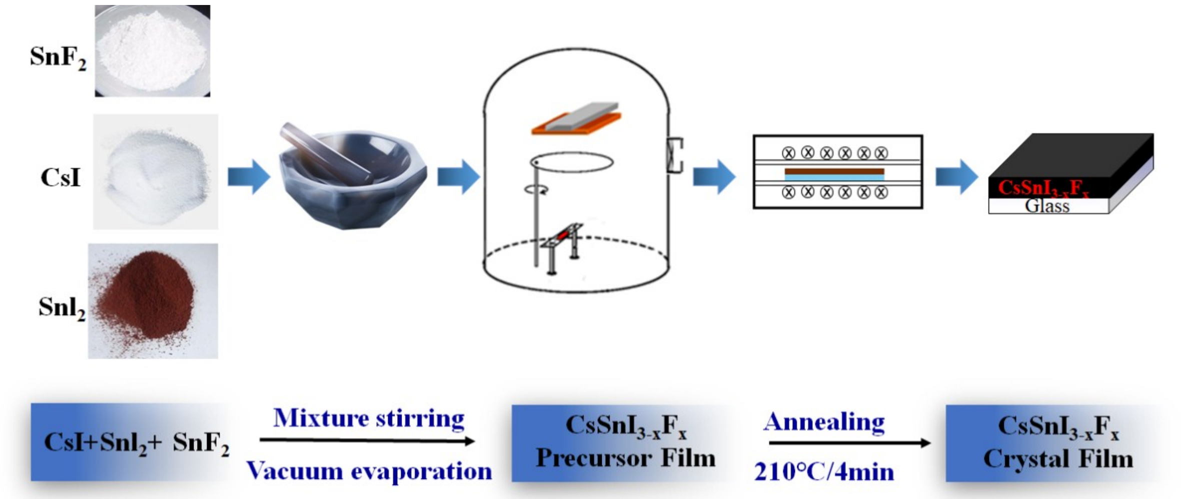

Fig.S1. Schematic illustration of the evaporation method.

To better understand of the role of F on the improved stability of $CsSnI_{3-x}F_x$ films, we decided to explore density functional theory (DFT) simulation using Vienna ab initio simulation package (VASP).

Supercells and F doping models of B-γ-$CsSnI_3$

B-γ-$CsSnI_3$ has 20 atoms and exhibits an orthorhombic structure with the *Pnma* space group symmetry. The supercells and F doping models corresponding to the minimum energy was identified as the computational model. Within the two supercells, the minimum energy of F doping models formed from the supercell A, and there are two doping positions: the horizontal and vertical positions.

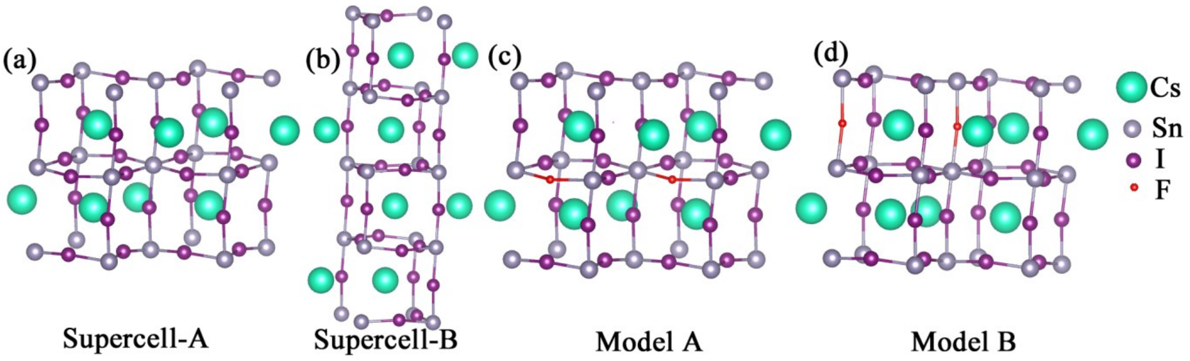

Fig.S2. Supercells and F doping models of B-γ-$CsSnI_3$, Cs is in green, Sn is in gray, the I anions are purple, and F is red.

Density of Localized Trapped Charge

The part of the content is quoted here: the local charge density in order to reveal the characteristics of bonding in the $CsSnI_{3-x}F_x$ films. Fig.S2 further presented the local charge density of B-γ-$CsSnI_{3-x}F_x$ films unit cells which x = 0, 1, 2 and 3. F-Sn-I clusters are more electronegative than I-Sn clusters because of that fluorine has strong electron binding ability. With the increase of fluorine content, Sn-I-Sn structure tends to I-Sn structure, that is to identify I turns from bidirectional to unidirectional, which explains the transition of Sn^{2+} to Sn^{4+} valence. It is clear shown that doping only plays a role of local stability. Thus, it is proven that the phase transition time is slowed down, not stopped. These results are consistent with our assumptions in **Introduction**.

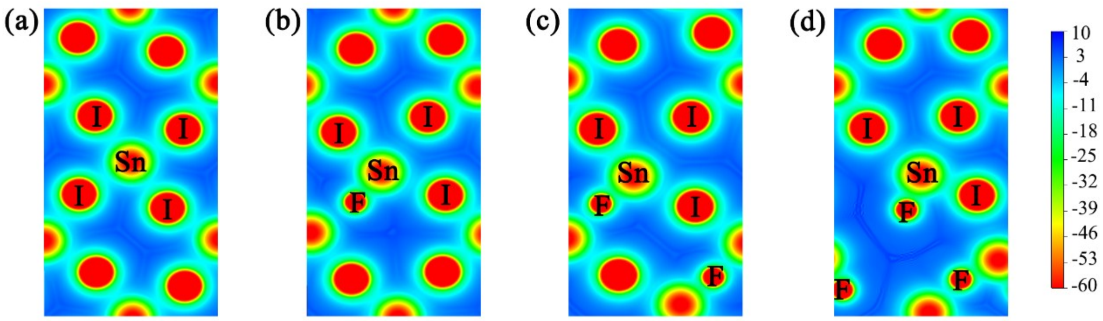

Fig.S3. Electron Localization Function for B-γ-$CsSnI_{3-x}F_x$ where x = 0, 1, 2 and 3. Blue regions are domains of high electron localization while red regions are domains of low electron localization.
